# TILLING in the two-rowed barley cultivar 'Barke' reveals preferred sites of functional diversity in the gene *HvHox1*

**DOI:** 10.1186/1756-0500-2-258

**Published:** 2009-12-17

**Authors:** Sven Gottwald, Petra Bauer, Takao Komatsuda, Udda Lundqvist, Nils Stein

**Affiliations:** 1 Leibniz Institute of Plant Genetics and Crop Plant Research (IPK), Corrensstr. 3, D-06466 Gatersleben, Germany; 2 Saarland University, Dept. Of Biosciences-Botany, Campus A2.4, D-66123 Saarbrücken, Germany; 3 National Institute of Agrobiological Sciences (NIAS), Plant Genome Research Unit, Kannondai 1-2-1, Tsukuba, Ibaraki 305 8602, Japan; 4 Nordic Genetic Resource Centre, P.O. Box 41, SE-230 53 Alnarp, Sweden

## Abstract

**Background:**

The economic importance of cereals such as barley, and the demand for improved yield and quality require a better understanding of the genetic components that modulate biologically and commercially relevant traits. While *Arabidopsis thaliana *is the premiere model plant system, the spectrum of its traits cannot address all of the fundamental questions of crop plant development. Unlike *Arabidopsis*, barley is both a crop and a model system for scientific research, and it is increasingly being used for genetic and molecular investigations into the conserved biological processes of cereals. A common challenge in genetic studies in plants with large genomes arises from the very time-consuming work of associating mutant phenotypes with gene sequence information, especially if insertion mutagenesis is not routine, as in barley. Reverse genetics based on chemical mutagenesis represents the best solution to this obstacle.

**Findings:**

In barley, we generated a new TILLING (Targeting Local Lesions IN Genomes) resource comprising 10,279 M_2 _mutants in the two-rowed malting cultivar 'Barke,' which has been used in the generation of other genomic resources in barley (~150,000 ESTs, DH mapping population). The value of this new resource was tested using selected candidate genes. An average frequency of approximately one mutation per 0.5 Mb was determined by screening ten fragments of six different genes. The ethyl methanesulphonate (EMS)mutagenesis efficiency was studied by recording and relating the mutagenesis-dependent effects found in the three mutant generations (M_1_-M_3_). A detailed analysis was performed for the homeodomain-leucine-zipper (HD-ZIP) gene *HvHox1*. Thirty-one mutations were identified by screening a 1,270-bp fragment in 7,348 M_2 _lines. Three of the newly identified mutants exhibited either a six-rowed or an *intermedium*-spike phenotype, and one mutant displayed a significantly altered spikelet morphology compared to that of the 'Barke' wild type. Our results indicate a bias in the frequency of independent functional mutations at specific base pair (bp) positions within the gene *HvHox1*.

**Conclusions:**

A new TILLING population was developed as a resource for high-throughput gene discovery in an alternative barley germplasm. Pilot screening demonstrated a similar or even slightly higher mutation frequency when compared to previously published barley TILLING populations that should allow for the identification of diverse allelic variation. Partial phenotypic evaluation of the M_2 _and M_3 _generations has revealed the presence of a wide spectrum of morphological diversity that highlights the great potential of this resource for use in forward genetic screens. Altogether, our study shows the efficiency of screening and the applicability of the new TILLING population for genetic studies in the barley crop model system.

## Background

Barley has a long history as a model plant in mutation research and breeding [1]. One year after Muller published his pioneering work on mutations induced by X-rays in fruit flies [2], Stadler published the first results on induced mutations in barley [3]. In the same year, the Swedish barley mutation research programme was initiated to study the fundamental effects of different mutagens on the barley genome and their applicability for breeding purposes [4]. Since spontaneous mutations occur with extremely low frequency, mutation induction techniques were widely expected to rapidly increase variability in crop species. After initial attempts, more than ten years passed before the first practical mutation procedures were implemented leading to improved mildew resistance in barley [5-7]. In 1959, a conference at today's Leibniz Institute of Plant Genetics and Crop Plant Research (IPK) substantially promoted interest in chemical mutagenesis [8]. Today, over 300 barley varieties are listed that were either directly or indirectly derived from mutation breeding [9], reflecting the impact of mutation induction on crop improvement.

In the past, the systematic development of mutagenesis was limited by the lack of effective mutation screening techniques and the rather basic knowledge of genes underlying the designated traits, rather than by the efficiency of mutation induction [10]. The introduction of TILLING (for Targeting Local Lesions IN Genomes) [11], which combines chemical mutagenesis with high-throughput genome-wide screening for point mutations in genes of interest, effectively complements classical forward mutation screening based solely only on phenotype. Chemical mutagens like ethyl methanesulphonate (EMS) induce high-density single nucleotide changes that are randomly distributed in the genome [12]. These DNA-damaging agents induce allelic variation that includes missense mutations in coding sequences (leading to altered protein structure and function), mutations of non-coding sequences that may affect gene regulation, as well as premature stop codon mutations and splice site changes that may result in knockout/knockdown of a protein. TILLING introduces new aspects into plant breeding, such as the possibility of surveying specifically for allelic series of economically interesting genes [13,14]. Moreover, as a highly precise and targeted approach, TILLING does not involve the generation of genetically modified plants, and hence it is a more globally accepted tool for breeding.

In the present study, we describe a new EMS-induced mutant population comprising 10,279 M_2 _individuals of the two-rowed spring-type barley cultivar 'Barke.' TILLING was used for ten DNA fragments derived from six different genes in order to estimate the average mutation frequency for this population. A more detailed analysis was performed for the gene *HvHox1*, which was previously shown to control the row-type character in barley [15], allowing for a frequency bias for functional mutation sites to be determined. Our results suggest that the new TILLING resource of the barley cultivar 'Barke' has potential for use in fundamental research as well as for applied breeding.

## Results

### Generation of a TILLING population in the barley cultivar 'Barke'

The two-rowed barley cultivar 'Barke' was used to develop a mutant population. The response to treatment with ethyl methanesulphonate (EMS) had to be determined first, because the genotype-dependent sensitivity of 'Barke' to chemical mutagenesis was unknown. Mutagenic efficiency was used as the method of validation. Based on previous experience with EMS mutagenesis with TILLING in a different barley cultivar ('Optic' [16]), nine EMS concentrations between 20 (0.2%) and 60 mM (0.63%) were applied. Relatively narrow increments of 5 mM were chosen since chemical mutagens are characterised by specific threshold values, after which the survival rates may quickly decline [17]. The mutation efficiency was monitored as the relative rate of mutation frequency in comparison to the frequency of undesirable changes at a given mutagen concentration. The applied range of concentrations was expected to allow differentiation between the most efficient as well as the maximum tolerated EMS treatment for the cultivar 'Barke.'

As a first parameter, the tolerance of 'Barke' against EMS toxicity was analysed by comparing the relative fitness as a function of the applied EMS concentration; the assessment was based on the respective frequencies of germination, sterility and M_2 _seed yield (Table 1). Germination rates significantly decreased from 92% for 20 mM EMS to an average of 40.5% for 60 mM EMS. In the same range of EMS concentrations, M_1 _spike sterility was scored in three categories: completely sterile spike (100% sterility), semi-sterile spike (measured as > 50%, 50%, < 50% sterility) and fully fertile spike (0% sterility). At increasing EMS concentrations, a clearly significant negative relationship was observed with M_1 _spike fertility leading to nearly 100% sterile spikes at 60 mM EMS (Table 1). For practical reasons in the context of population development, treatment with 40 mM EMS was found to be the upper limit, as the level of fully sterile spikes increased from 63% at 40 mM to 84% at the next higher concentration of 45 mM. Consequently, the overall number of M_1 _plants carried to the next generation and that were used to build up the overall population varied substantially between treatments (Table 2). In summary, from 99,879 mutagenised barley seeds (M_0_), 24,600 M_1 _plants advanced to the M_2 _generation.

**Table 1:** Average germination and sterility rates (%) of M_1 _plants observed after different EMS Treatments

	F^d)^	EMS treatments									
		0 mM	20 mM	25 mM	30 mM	35 mM	40 mM	45 mM	50 mM	55 mM	60 mM
germination^b)^	6.1**	-	92	89	81	75.3	69.3	59.7	56.7	49	40.5
full sterility^c)^	41.7***	4	3	21	26	30	63	84	87.7	87.5	95.5
semi-sterility^c)^	17.5***	63	81	74	83	68	37	16	13	12	5
no sterility^c)^	8.97***	33	16	5	2	1.8	0.6	0.06	0	0	0

^a) ^In total 72,879 M_1 _plants were cultivated in 299-well seed trays on flood tables.

^b) ^All germination data were collected as percentages of germination rates observed for the 0 mM control plants.

^c) ^Sterility was scored for the examined M_1 _spikes in three categories: fully sterile (100%), semi-sterile (measured as > 50%, 50%, < 50%), or no sterility (0%).

^d) ^The one factorial analysis of variance (ANOVA) tested the variability within each EMS concentration and between all concentrations. ANOVA: F = Fisher's exact test: *: Significance at p ≤ 0.05; ** Significance at p ≤ 0.01; *** Significance at p ≤ 0.001. A test of linearity was performed to confirm the required normality of the single data set (data not shown) [63].

**Table 2:** M_2 _population structure: number of M_2 _lines contributing to the population from different EMS concentrations

EMS treatment	20 mM	25 mM	30 mM	35 mM	40 mM	45 mM	50 mM	55 mM	60 mM	Total
Final population	1,949	2,900	2,622	1,965	469	130	161	51	32	10,279
Population used for TILLING	1,949	1,297	2,451	1,101	401	-	107	51	32	7,389

Plant fertility was also assessed in the M_2 _generation since efficient seed set in M_2 _is a prerequisite for effective phenotyping of M_3 _families identified after TILLING. The M_2 _seed yield was negatively correlated with EMS dosage (r = -0.93 and r = -0.99). From the 20 mM-treated M_2 _mutants, on average more than 90 seeds were harvested regardless of whether the plants were grown under greenhouse or field conditions. Only 65% of the plants receiving 40 mM EMS produced M_3 _seeds, and 38% of the plants with the highest EMS concentration yielded M_3 _seeds that could be successfully harvested (data not shown).

Based on prior experience with reduced fertility in advanced mutant generations, ten plants per M_2 _family were initially cultivated from 15,346 M_1 _plants to construct the final TILLING population. After tillering, the M_2 _families were evaluated for viability, and one M_2 _individual from each family was selected for further cultivation and subsequent development of M_2 _DNA and M_3 _seed stocks. This selection was done in order to avoid redundancy of mutation events in the final population. For barley, it was assumed that only one primordial germ line cell of the embryonic main shoot apex was present in M_1 _[4]. Therefore, M_1 _seeds of a single spike could share the same mutational event, and a single mutation could appear as a cluster in the segregating spike progenies [18]. The main selection criterion at this stage of population development was general vitality (reduced risk of growing sterile plants), since high yields of M_3 _seeds were targeted as an essential prerequisite for the development of a useful and sustainable TILLING resource. In some cases (1.7% of all M_2 _families), when no clear decision could be made for the selection of a single M_2, _two plants of the same M_2 _family were kept until maturity. Such siblings were further handled as independent mutant line accessions. Finally, a set of 10,279 M_2 _plants derived in different proportions from various EMS concentrations (Table 2) was selected to construct the overall population.

For the efficient development of a mutant population, it is desirable to get the earliest possible assessment of the achieved mutation density. Since results of previous barley mutagenesis experiments revealed the correlation between EMS dosage and M_2 _chlorophyll seedling mutant frequencies [19], M_2 _families were evaluated for such early phenotypic markers. The analysis focused on the five most frequent phenotypes: *albina, xantha, viridis *(all three belonging to sub-class unicolour [20]), *viridoalbina *and *striata *(both belonging to sub-class bicolour [20]) (Additional file 1). The frequency of such phenotypes was determined in progeny obtained at EMS concentrations ranging from 20 to 50 mM (Table 3). The relative frequencies were calculated based on the number of mutant individuals per 100 germinated M_2 _plants. Although this method does not consider cluster mutations within the M_2 _families, compared to spike progeny-based methods [20] it has the advantage of being insensitive to variations in the respective spike progeny sizes, which are affected in M_2 _families especially at higher EMS treatments (data not shown). The *albina *phenotype was most frequently observed (Table 3), and a significant relationship between EMS treatment and frequency of chlorophyll-deficient mutants was exclusively seen for this phenotype. The other four investigated phenotypes seemed to be distributed randomly, suggesting that those mutations might be conditional and vary depending on the environment, similarly to the results seen for several chlorophyll seedling mutants in previous studies [21,22].

**Table 3:** Average M_2 _frequencies of chlorophyll seedling mutants (%) after different EMS treatments

Classification^a)^	Phenotype	F^b)^	EMS treatment					
			20 mM^1)^	25 mM^2)^	30 mM^3)^	35 mM^4)^	40 mM^5)^	50 mM^6)^
Unicolour	albina	11.8***	1.5	2.3	3.4	4.3	6.6	7.0
Unicolour	xantha	0.61	0.2	0.6	1.2	0.4	1.5	-
Unicolour	viridis	1.10	0.1	0.4	0.6	1.3	0.5	-
Bicolour	viridoalbina	1.62	0.4	0.2	0.4	0.8	0.5	-
Bicolour	striata	1.08	0.15	0.1	0.3	0.9	0.5	-

Relative frequencies were calculated as mutations per 100 germinated M_2 _plants according to [20]. Number of analysed individuals: ^1) ^17,616; ^2) ^13,881; ^3) ^20,820; ^4) ^14,190; ^5) ^1,270; ^6) ^719. Chlorophyll mutant rates other than albina observed among M_2_seedlings obtained from treatments higher than 40 mM were excluded from statistical considerations because the generally low germination rate of those mutants was insufficient.

^a) ^Chlorophyll seedling mutant classification according to [20].

^b) ^The one factorial analysis of variance (ANOVA) tested the variability within each EMS concentration and between all concentrations. ANOVA: F = Fisher's exact test: *: Significance at p ≤ 0.05; ** Significance at p ≤ 0.01; *** Significance at p ≤ 0.001. A test of linearity was performed to confirm the required normality of the single data set (data not shown) [63].

### Screening for morphological mutants (forward genetics) in the TILLING population

In total, 12,703 M_2 _individuals (EMS dosages between 20 and 35 mM) were scored for their phenotype during field (11,661 M_2_) or greenhouse (1,042 M_2_) cultivation. All plants were observed at two-week intervals from the seedling stage to full maturity with specific emphases on altered spike morphology, heading date (and plant development in general), plant colour, plant height, and other obvious variations form the 'Barke' wild-type. About 20% of the surveyed M_2 _plants displayed noticeable phenotypes, which were classified into six morphological categories (Additional file 2A). The most common category, plant height (Additional file 2A), contained 45% of all visible phenotypes and mainly comprised all different kinds of dwarfism. Among the 238 M_2 _plants classified as plant colour mutants, 21% (50 of 238) were impaired in surface wax structure, including such types as *glossy sheath *and *eceriferum*. Another 18% (43 of 238) were assigned as viable *Chlorina *seedlings, showing light- or yellow-green leaves and a stunted growth habit, and these comprised the second most frequent type of the plant colour mutant category.

In addition to phenotypic screening in M_2_, 1,200 M_3 _families (derived from 20 to 40 mM EMS treatment) were screened as batches of 1,000 and 200 families, respectively, under field conditions from the subsequent years 2006 and 2007. Screening and classification were carried out as described for the M_2 _population (Additional file 2B). Approximately 37% of all analysed M_3 _families showed a mutant phenotype; of these, 5.6% were homozygous for the observed trait, and another 5.6% of the families were segregating two independent phenotypes (data not shown). In 2.8% of the families, plants were observed that displayed more than one aberrant phenotype that could be ascribed to known monogenic mutations (data not shown). In contrast to the phenotypic spectrum observed in the M_2 _generation, alterations of tillering and plant colour were observed more frequently, while the frequency of dwarfism was notably lower (Additional file 2B).

The frequency of visible morphological phenotypes correlated positively with EMS dosage. In the M_2 _generation, this frequency ranged from 10% among 20 mM-treated plants to 23% among plants receiving 35 mM EMS. By contrast, in the M_3 _population, this ranged from 18% among 20 mM-treated plants to 63% among plants receiving 35 mM EMS.

### Determination of molecular mutation frequency--TILLING of candidate genes (reverse genetics)

In order to survey the mutation frequency of the composite population at the molecular level, ten fragments from six genes were screened. This work was performed in parallel to the late stages of population development, so only a limited set of 7,389 M_2 _lines could be used for TILLING (Table 4) (except for the gene *mlo *for which only 1,920 M_2 _were surveyed). The mutation frequency per gene fragment was estimated by dividing the total base pairs screened by the total number of mutations identified. The density of mutations was calculated in the established barley population as an average frequency of all screened fragments. Scoring of mutations located either in the proximal or distal 50 bp of each amplicon was compromised because of priming and systematic artefacts on the LiCOR gels. Therefore, in order to calculate the effective number of base pairs screened, each amplicon length was reduced by 100 bp. Gene fragments ranging from 321 bp to 1,400 bp in length were designed to cover either whole open reading frames (ORFs) or selected coding regions of the respective target genes.

**Table 4:** List of TILLING targets, size of amplicons, and the number and distribution of mutation types

Gene	Description	Amplicon	Amplicon size (bp)^1)^	Mutation					Frequency (Mb)^5)^
				total	Intron	syn^2)^	non-syn^3)^	trun^4)^	
*HvCO1 *^6)^	CONSTANS-like gene	1	830	8	-	5	3	-	1/0.67
*Mlo9 *^7)^	Modulator of powdery mildew resistance and cell death	1	900	2	-	-	1	1	1/0.75
		2	1476	3	1	2	-	-	1/0.87
*HveIF4E *^8)^	eukaryotic translation initiation factor 4E	1	367	7	3	-	4	-	1/0.28
		2	321	4	2	-	2	-	1/0.41
*HvDnaJ-like *^9)^	DnaJ-like chaperone	1	434	7	2	2	3	-	1/0.36
*HvHox1 *^10)^	Homeodomain transcription factor controlling row-type morphology in barley	1	985	22	7	6	8	1	1/0.3
		2	362	9	2	5	2	-	1/0.2
*HvCIGR2 *^11)^	Barley homolog of rice 'Chitin-inducible gibberellin-responsive'	1	699	13	-	10	2	1	1/0.34
		2	702	6	-	2	4	-	1/0.63

^1) ^7,389 M_2 _lines were screened for mutations in the listed genes, except for the *Mlo9 *gene that was analysed in 1,920 M_2 _lines. ^2) ^syn = synonymous mutations that do not alter the AA sequence of the protein; ^3) ^non-syn = non-synonymous mutations that introduce a change of AA in the protein sequence; ^4) ^trun = truncation mutations that generate a premature stop codon or alter a splice junction, potentially resulting in a truncation of the protein; ^5) ^The mutation frequency for each amplicon is calculated as follows: (size of amplicon --100 bp × total number of screened samples)/total number of identified mutations; 100 bp were subtracted because of the diminished ability to detect mutations in the upper and lower 50 bp; ^6) ^[64]^7) ^[65,66]^8) ^[56]^9) ^NCBI accession BQ470183 ^10) ^[15]^11) ^NCBI accession EU914128 (bp position 69,100-70,440)

The screen revealed a total of 81 independent mutations (Table 4), including 64 (79%) located in exons. Twenty-nine (45%) of the exon-located mutations induced a change of amino acid (AA) sequence, and three (5%) affected splice sites or inserted a premature stop codon, which most likely led to truncated reading frames. Based on all of the screened fragments, the average mutation frequency for the population was found to be one mutation per 0.5 Mb. Therefore, the entire population of 10,279 M_2 _lines translates to an average of 20 mutations for any 1 kb DNA fragment. The probability of having at least one truncation mutation is about 60% [1-(1-0.04)^20^; based on the overall truncation frequency of 4% (3/81)].

**Figure 1:**
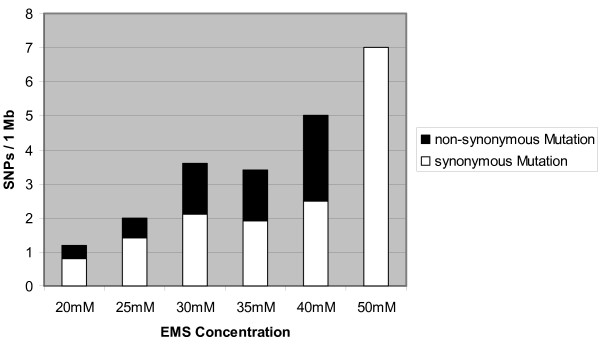
**Mutation frequency in relation to EMS concentration**. The relative SNP rates for EMS treatments between 20 and 50 mM were calculated as particular sub-population specific frequencies. Data are based on the mutation screening of eight gene fragments in 7,389 M_2 _lines. The white and black bars show the accumulated synonymous and non-synonymous (AA changes and truncation) mutations detected for each sub-population. The relationship between SNP frequencies and EMS dosages was calculated based on a non-parametric Spearman rank correlation (coefficient of r_s _= 0.94 with a significance at p ≤ 0.01).

Since the TILLING population was derived from seed batches treated with different EMS concentrations, the results could be correlated to EMS dosage [mutation frequency _[EMS] _= ∑ SNPs per gene/∑ screened Mb per gene]. The single base pair substitution rate was positively correlated (r_s _= 0.94) with increasing EMS concentration (Figure 1) and ranged (based on TILLING of 8 PCR-derived DNA fragments from 5 genes) from 1 mutation/0.8 Mb at 20 mM EMS to 1 mutation/0.20 Mb and 1 mutation/0.14 Mb after treatment with 40 and 50 mM EMS, respectively. Likewise, an increase in the respective levels of missense mutations was observed between 20 mM- and 40 mM-plants, while there were not any non-synonymous mutations observed with the highest treatment (Figure 1).

### New alleles and location bias of functional mutations in the gene *HvHox1*

A detailed analysis was performed for the *HvHox1 *gene (homeodomain-leucine-zipper (HD-ZIP) containing transcription factor), which has been shown to encode the row-type controlling locus *Vrs1 *in barley [15]. Wild-type barley has a two-rowed ear morphology where only the central of three sessile spikelets is fertile and produces seeds. The recessive allele *vrs1 *affects the fertility of the lateral spikelets, leading to a six-rowed ear. The cultivar 'Barke' possesses a two-rowed ear morphology, so TILLING has the potential to survey for *de novo *functional alleles with six-rowed phenotypes.

**Figure 2:**
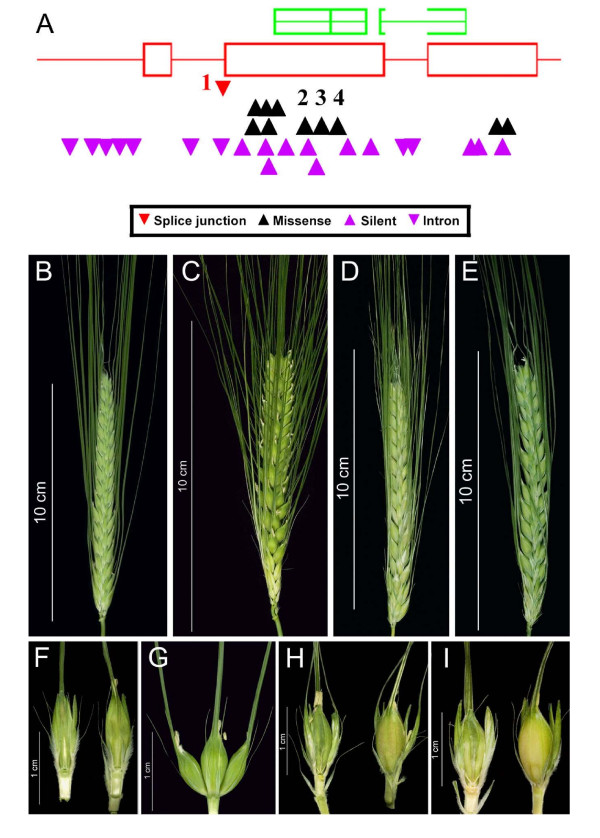
**Tilling of the gene HvHox1 reveals new alleles of the two-rowed/six-rowed locus Vrs1**. **(A) **Model of the gene *HvHox1 *(*Vrs1*) designed from output files of the software PARSESNP [58]. Gene structure and distribution as well the type of mutation discovered in a 1,270-bp region covering the entire open reading frame (ORF) of the gene are illustrated: exons = red open boxes, introns = red lines, conserved homeodomain-leucine zipper I-class homeobox domain (obtained from the BLOCKS database, [67]) = green boxes above the gene structure model. Mutations are indicated as small arrowheads below the gene structure model: purple = non-functional mutations, i.e., intron-located or synonymous mutations; black = non-synonymous mutations; and red = a splice junction mutation. Numbered arrowheads indicate mutations leading to phenotypic changes in M_2 _individuals 11910-1 (1), 3930-1 (2), 8408-1 (3), and 11657-1 (4), respectively. Detailed views of total inflorescences **(B-E) **and lateral spikelet morphology **(F-I) **are given for wild-type (two-rowed) 'Barke' **(B, F)**, a six-rowed (*hex-v*) mutant (8408-1) **(C, G)**, an *Intermedium spike-d (Int-d) *mutant (11657-1) **(D, H)**, and mutant 3930-1 **(E, I)**. Mutant 8408-1 possessed fully fertile and awned lateral spikelets **(G)**, whereas **the **lateral spikelets of mutant 11657-1 exhibited shorter awns and only partial fertility, mostly in the basal lateral spikelets **(H)**. The lateral spikelets of 3930-1 were significantly enlarged and tip-pointed compared to 'Barke,' but were sterile and not awned **(I)**.

A 1,270-bp region covering the entire open reading frame (ORF) of *HvHox1 *was screened in 7,348 M_2 _lines by analysing two overlapping fragments, giving a total of 9.4 Mb of gene sequence analysed. Thirty-one alleles, including ten missense mutations and one splice junction mutation, were identified (Additional file 3). The majority of these mutations (25 of 31) were GC to AT transitions, and 68% (21 of 31) of all mutations in *HvHox1 *were found in the homozygous state. The average mutation frequency was 1 mutation/0.25 Mb (Additional file 3; Figure 2A). A comparable departure from the expected 1:3 homozygous mutant versus heterozygous and homozygous wild-type ratio for M_2 _plants was reported by TILLING in the barley cultivar 'Morex,' and it was interpreted as a possible sensitivity limitation for the detection of heterozygotes in 8-fold DNA-pools [23]. Seven missense mutations induced changes to AAs with altered physicochemical properties in regards to polarity and/or hydrophobicity. Four of the missense mutations were detected in the conserved and functionally relevant homeodomain-leucine zipper motif (HD-ZIP).

For all of the M_2 _plants that exhibited a non-synonymous mutation, 16 to 20 M_3 _individuals were grown for phenotyping of the row-type character. The progeny of each line were subsequently genotyped. In three of the four missense mutations affecting the HD-ZIP domain, a change in spike appearance, especially in the lateral spikelets, was observed (Figure 2). Mutant line 3930-1, which carried a G to A transition at the 1,039-bp position, exhibited significantly enlarged [ANOVA F-values of 334*** (spikelet length) and 614*** (spikelet width) at a significance value of p ≤ 0.001] and tip-pointed but sterile lateral spikelets without awns in the M_3 _progeny (Figure 2E, I). The mutant line 8408-1 (T1079A) showed a *hexastichon *(*hex-v*) six-rowed phenotype (Figure 2C, G), and the mutant 11657-1 (G1115A) displayed an *Intermedium-d spike *(*Int-d*) phenotype (Figure 2D, H). Mutant 11657-1 was the only case where a conservative substitution (Arg to His) led to a visible impact. Finally, a second *hexastichon *(*hex-v*) six-rowed phenotype was obtained from mutant line 11910-1 carrying a splice junction mutation at the 3' end of intron 1.

**Figure 3:**
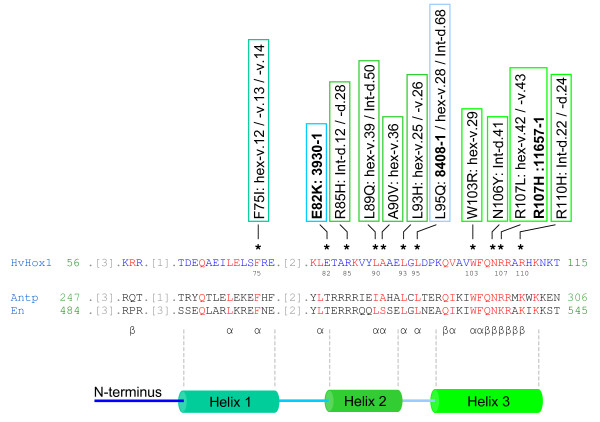
**Amino acid conservation in the HvHOX1 homeodomain**. AA conservation in the HvHOX1 homeodomain was assessed by a sequence comparison of 100 different homeodomains (homeodomain smart accession No. 00389) provided by NCBI's Conserved Domain Database (CDD) [61,62]. Highly conserved AAs are highlighted in red, whereas less conserved AAs are highlighted in blue. Positions of the AAs in each protein are indicated with numbers flanking the corresponding peptide, and AAs are given in single letter code. Asterisks above the HvHOX1 sequence indicate induced and non-synonymous substituted AAs detected in 21 barley mutants. The corresponding mutant lines together with the respective AA substitutions are shown in the boxes above. Mutations obtained from the 'Barke' mutant population are depicted in bold letters. The remaining mutations originate from alleles of the cultivars 'Bonus,' 'Foma' and 'Kristina,' as previously described by [15]. The homeodomains of the proteins Antennapedia (Antp) (*Schistocerca americana*, American grasshopper) and Engrailed (En) (*Drosophila virilis*) were used as references for the localisation of the functionally relevant AAs in HvHOX1, as previously described [29-32]. The main structural features are indicated by α, core AAs involved in the determination of the three-dimensional protein structure; and β, AAs implicated in DNA binding. A model of the secondary structure of the HD peptide is indicated below the alignment, beginning with the N-terminus.

All phenotypically relevant missense mutations were found in the functionally important homeodomain (HD) region of the HvHOX1 protein, while mutants with AA changes outside the HD-ZIP domain displayed normal two-rowed spikes. The four *HvHox1 *mutants newly identified in the 'Barke' TILLING population that displayed a mutant phenotype were compared to a set of 18 missense alleles previously induced and reported for the cultivars 'Bonus,' 'Foma,' and 'Kristina' [15]. Among all 22 mutant lines, 9 of the 21 highly conserved AAs of the homeodomain domain were affected (Figure 3).

Some of the new 'Barke' TILLING mutations coincided with AA substitutions in previously induced *vrs1 *alleles (Figure 3). A guanine to adenine transition was found at bp position 1115 in mutant 11657-1, leading to a change from Arg to His. The same nucleotide was affected by a G to T transversion (Arg substituted by Leu) in the mutant lines *hex-v.42 *and *43 *induced in the cultivar 'Kristina' by fast-neutron and EMS treatments, respectively [15].

'Barke' mutant 8408-1 shared an A to T transversion at bp position 1,079 (L95Q) with previously identified mutants *hex-v.28 *(ethylene imine (EI), cultivar 'Foma') and *Int-d.68 *(EMS, cultivar 'Kristina') [15]. All three of these mutant lines carried an identical AA substitution, raising the question of whether the newly observed 'Barke' allele was induced independently or if it originated from pollen/seed contamination by a common allele of six-rowed barley cultivars. To test this, we analysed the entire DNA sequence of the 'Barke' *Vrs1 *allele. Instead of having one of the three haplotypes *vrs1.a1*, *vrs1.a2 *and *vrs1.a3 *common for modern six-rowed cultivars, the *HvHox1 *gene of the mutant 8408-1 exhibited the haplotype of the two-rowed genotype 'Barke' (except for the transversion at bp position 1,079). Furthermore, we never observed any six-rowed plant among the > 90,000 M_1 _'Barke' plants, supporting the hypothesis of a newly derived allele. Furthermore, a 'Barke' genetic background was confirmed for 8408-1 by genotyping 25 EST-based SSR markers (data not shown). Altogether, we were able to rule out that the allele of 8408-1 was due to any kind of contamination and to confirm that the 8408-1 allele was derived from 'Barke' by EMS mutagenesis

The alleles of the mutants 3930-1 and 11910-1, which lead to enlarged pointed lateral spikelets or a true *hex-v *phenotype, respectively, also turned out to be novel *vrs1 *alleles, as they involved previously unaffected AAs and nucleotide positions. Interestingly, the splice-junction mutation of 11910-1 (G856A) was separated only by 1 bp from a previously described mutation at bp position 855 that was induced in the genotype 'Kristina' by isopropyl methanesulphonate, leading to mutant hex-v.38.

## Discussion

Genetic mutation is a powerful tool that establishes a direct link between the biochemical function of a gene product and its role *in vivo*. For decades, genes have been identified and functionally characterised by the isolation and study of mutants that are defective in a specific process of interest. With the advent of molecular biology, several reverse genetic techniques have been developed for the functional characterisation of cloned genes. In plants, the most commonly used reverse genetic approaches are post-transcriptional gene silencing (PTGS) [24] and insertional mutagenesis [25]. However, PTGS is labour-intensive and can give ambiguous results. For insertional mutagenesis, the frequency of mutations per genome is typically low, and the mutant alleles are likely to result in a complete loss-of-function of the gene, which might limit the analysis if the effect is lethal or detrimental. By contrast, the TILLING technology induces diverse allelic variation, including missense mutations that can lead to altered protein function, as well as premature stop codons and splice site changes that can result in knockout or knockdown of a gene [26].

TILLING of the homeodomain-leucine-zipper (HD-ZIP) gene *HvHox1 *[15] in our newly developed 'Barke' cultivar TILLING population demonstrated the feasibility of generating an allelic series of a gene in barley with subtle phenotypic variation. In our study, one splice-junction mutation and three missense mutations were identified that provided three different types of mutant spike morphology. Almost all phenotypically relevant missense mutations of this and previous studies [15] were found to be situated in the functionally relevant homeodomain (HD) region. Those mutants displaying a full or intermediate six-rowed phenotype affected AAs that are universally conserved within members of the HOX protein family [27]. The different mutant phenotypes might be explained not only by the distinct positions of the substitutions in the protein sequence, but also by the character of the amino acid replacement. HOX proteins are DNA-binding transcriptional regulators that contain a highly conserved homeodomain, which is characterised by three α-helical regions (helices 1 - 3) that fold into a tight globular bundle to form a so-called helix-turn-helix (HTH) motif [28]. The AAs of the HvHOX1 homeodomain with putative functional relevance can be predicted by referring to the ANTENNAPEDIA (Antp; AAB03236) and ENGRAILED (En; XP_002050130) protein sequences, a method applied in previous studies [29-32]. The arginine at position 107 (Arg107), which is predicted to be involved in direct DNA binding by water-mediated hydrogen bonds between the homeodomain and DNA [31,32], was affected in two previously identified mutants as well as in one of the 'Barke' mutants. In all three cases, the mutation led to phenotypic changes, but of varying severity. The *hex-v.42 *and *hex-v.43 *mutant alleles (originating from the 'Kristina' cultivar) were altered in hydrophobicity (Arg to Leu) and displayed a fully six-rowed phenotype (Takao Komatsuda, unpublished data). A conservative substitution (Arg to His) was identified in the 'Barke' mutant 11657-1, and this led to an *Intermedium spike-d *(*Int-d*) phenotype with less developed, short-awned and fully sterile lateral spikelets.

Nine of the 21 (43%) strictly conserved AAs of the homeodomain of *HvHox1 *were affected by substitutions in previously analysed phenotypic mutants [15]. From the 'Barke' population, we obtained a total of nine mutants that were affected in this region; two mutants, 11657-1 and 8408-1, contained mutations at AA positions identical to induced alleles previously published. This coincidence was surprising but not necessarily unexpected. The probability of detecting a mutation at the same AA of at least one of the previously found substitutions is 99% [1-(1-0.43)^9^].

The study of mutants originating from different germplasms can provide added value in the functional analysis of a trait or gene. However, it needs to be kept in mind that phenotypic differences of mutants at the same position of the gene may also be the result of modifying loci. Row-type in barley is controlled by the major gene *vrs1 *(*HvHox1*), but interaction with the unlinked *intermedium *(*int)-c *locus has been demonstrated. Plants heterozygous for *vrs1 *exhibited different degrees of fertility and seed size in lateral spikelets within or between different six-rowed plants in the presence of different *int-c *alleles [33-35]. Therefore, even mutations in the same AA of the HvHOX1 protein can lead to different phenotypes depending on the *int-c *allele. As a consequence, the availability of TILLING resources in different germplasms can be important while studying specific traits. Our TILLING population was generated in the two-rowed central European malting variety 'Barke,' whereas previously published TILLING populations were established either in the two-rowed Scottish malting cultivar 'Optic' or the six-rowed North-American malting variety 'Morex' [16,23]. TILLING of HvHox1 in the latter population would not have been feasible.

Access to TILLING resources in different genetic backgrounds could also be of importance if mutant traits will be utilised in plant breeding. Every individual of a TILLING population typically accumulated a high density of mutations. Given an average mutation frequency of 1 mutation/0.5 Mb, a haploid genome size of 5,000 Mb for barley [36], and an overall number of 40,000 barley genes with an average ORF length of 2.5 kb [37,38] at an average G/C content of approximately 50% [39,40], the presence of approximately 100 induced SNPs that affect ORFs can be assumed for every plant of the TILLING population [Total SNP _in ORFs _= 40,000 ORFs × 1.25 kb^~G/Ccontent per ORF ^× ^1 SNP ^/_500 kb_]. Of these, about 40% will be non-synonymous mutations, and perhaps one fourth of these will be homozygous. Therefore, each M_2 _plant will theoretically contain at least ten profoundly affected genes per genome. This background load of mutations is often seen as a major obstacle for utilising TILLING-derived mutant alleles in plant breeding. The background mutational load needs to be removed by recombination in multiple generations of backcrossing or cross-breeding [41]. After backcrossing for six generations, theoretically only 1.5% residual heterozygosity will remain in a mutant, so the true efficiency of removing linked, undesired mutant variation may differ significantly depending on the region of the genome. Linkage disequilibrium (LD), the effect that closely linked genes will be inherited as haplotype blocks at higher than statistically expected probability, may extend to modern barley germplasms over distances of more than 50 cM [42,43]. Notably, LD is typically high in the centromeric regions of barley chromosomes where recombination frequency is low [44]. However, the impact of LD is not only relevant for predicting the efficiency of removing background mutational load. The presence of natural diversity in a detrimental modifier locus in LD to a desired mutant locus could have a similar effect. In such cases, the principle availability of induced allelic diversity in different genetic backgrounds may facilitate the use of TILLING mutants in a breeding program.

A high mutation frequency is of paramount importance for the development of a TILLING population because it is the key factor influencing the necessary effective population size and thus also the labour investment required for screening. A moderate mutation frequency of approximately one mutation per 0.5 Mb, which is characteristic of the present 'Barke' population as well as of previously published barley TILLING populations [16,23], requires a relatively large number of lines (~10,000) to achieve genome saturation. Therefore, access to barley TILLING populations featuring high mutation frequencies comparable to those reported for *Arabidopsis *[12,45] is an alluring prospect. Interestingly, during our efficiency analysis, we were able to demonstrate such high mutation frequencies for barley because we encountered levels of approximately one mutation per 0.2 and 0.14 Mb among mutants obtained from 40 and 50 mM EMS treatments. Although promising, the reproducibility of such high mutation frequencies needs to be confirmed in future experiments since the subpopulations obtained from 40 mM and 50 mM EMS concentrations were only 4% and 1.5% of the overall population, respectively.

Indeed, high mutagen dosages can impose practical problems, and improved screening efficiency may be compromised due to undesirable effects [46] since high mutagen dosage is typically associated with reduced fitness and fertility in advanced generations. In EMS-derived M_1 _plants, these effects are mainly caused by the toxicity of EMS metabolites, which leads to reduced M_1 _seedling germination, decreased growth and survival rates, and partially reduced M_1 _spike fertility. Over 80% fully sterile M_1 _'Barke' plants were obtained using EMS concentrations above 40 mM. Space constraints for raising a barley TILLING population are significantly higher compared to *Arabidopsis*; thus increased M_1 _and M_2 _sterility at high EMS concentrations cannot easily be compensated for by increasing the M_1 _population size. Therefore, building the final bulk of the 'Barke' TILLING population from treatments in the range of 25 - 40 mM EMS was driven by a compromise between effectiveness and practicality.

## Conclusions

We developed a TILLING population of 10,279 M_2 _plants as a resource for functional analysis of genes and for high-throughput gene discovery in an alternate germplasm of the crop model system barley. The screening of six target genes demonstrated a moderate mutation frequency of approximately one mutation per 0.5 Mb. This frequency should allow for the identification of multiple randomly distributed mutations in chosen gene regions. Phenotypic evaluation of portions of the M_2 _and M_3 _generations revealed the presence of a wide spectrum of morphological diversity, which is a further indication of the forward genetic potential of this resource.

A detailed TILLING analysis was performed for the homeodomain-leucine-zipper (HD-ZIP) gene *HvHox1*, which is the major factor controlling the row-type morphology of the barley spike. Multiple alleles causing phenotypic changes of the two-rowed spike morphology of the 'Barke' cultivar were obtained. A comparison of newly identified and previously characterised *HvHox1 *mutants indicated a bias for preferred nucleotide/AA positions that lead to altered row-type character if affected by mutation. These results contribute to a better understanding of the functionally relevant sites of the HvHOX1 protein. Furthermore, this exemplary case illustrates the specific potential of TILLING to provide multiple independent alleles of a single gene with varying levels of phenotypic expressivity.

## Methods

### Seed material

Seeds of *Hordeum vulgare *L. cultivar 'Barke' (obtained from: Saatzucht Josef Breun GdbR, Herzogenaurach, Germany) were used for chemical mutagenesis. 'Barke' is a spring-type, two-rowed, European malting variety. 'Barke' was generated by cross-breeding between the two-rowed spring barley varieties 'Alexis' (BSA 1102) and 'Libelle' (1256) in 1996. The pedigrees of both 'Barke' parents are: 'Alexis' ('Br1747' × 'Rupee') × 'Br1622'from 1990, and 'Libelle' ('Br 1622' × 'Trumpf') from 1968 (Saatzucht Josef Breun GdbR, personal communication).

### Chemical mutagenesis

Barley seeds were treated at different concentrations with the chemical mutagen ethyl methanesulfonate (EMS) according to David G. Caldwell (personal communication). Batches of ~1,600 seeds were used to fill 2000-ml glass flasks and were pre-soaked in 500 ml of deionised water (ddH_2_O) for 4 h at room temperature (20-25°C). Then, the water was replaced by 350 ml of an EMS solution [20-60 mM in ddH_2_O], and seeds were gently shaken (125 rpm, tabletop shaker) for 16 h at room temperature. Subsequently, the EMS solution was collected for decontamination. The seeds were washed two times with 250 ml of 200 mM sodium thiosulphate (30 min for each step) and subsequently two times with 1 l of ddH_2_0 (first for 30 min, second for 1 h). After removing the supernatant, the seeds were transferred to trays covered with Whatman paper and air-dried at 4°C (16 h) prior to sowing.

### Creation of the TILLING population

#### Cultivation of M_1_

M_1 _plants were greenhouse-grown until maturity in 299-well seed trays placed on tables that were flooded daily for 12 minutes. After heading, the flooding was reduced to a two-day cycle. Twenty-three and twenty-nine days after sowing, seedlings were sprayed with a fertiliser solution (0.2% Wuxal, nitrogen-phosphate-potassium 8-8-6, AGLUKON GmbH & Co. KG, Düsseldorf, Germany). Subsequently, plants were watered by flooding the tables every second or third day in the summer and winter, respectively, with water-soluble Hakaphos blue (nitrogen-phosphate-potassium 15-10-15 & nutrient salts, COMPO GmbH & Co.KG, Münster, Germany). Every M_1 _generation was evaluated for germination and sterility rates. Germination was measured as the percentage of developed seedlings, and sterility was measured as seed set per harvested main spike. Germination data were collected 8 days after sowing for EMS treatments between 20 and 40 mM and 12 days after sowing for concentrations between 45 and 60 mM (due to the delayed development of those seedlings). Plants were allowed to self-pollinate, and the main spike of each plant was harvested as a source of M_2 _seeds.

#### Cultivation of M_2_

From each M_1_, a maximum of ten M_2 _individuals was cultivated as a family to the tillering stage (BBCH 30, [47]). Subsequently, plants were evaluated, and one or two individuals of each family were selected based on viability to ensure a sufficient seed set for further cultivation. In cases of two selected individuals per family, the siblings were integrated into the population as individual mutant lines.

The M_2 _plants were cultivated as batches either under greenhouse (1,000 M_2 _families) or field (3,780 M_2 _families) conditions. The M_2 _seedlings were regularly monitored for the presence of chlorophyll defects and other visible mutant phenotypes. For the analysis of chlorophyll mutant frequencies, we calculated the relative frequencies as mutations per 100 germinated M_2 _plants [48]. All phenotypes were scored in reference to the parent cultivar 'Barke.' The M_3 _seeds from individual M_2 _mutants were collected, catalogued, vacuum-packed and stored at 4°C until use as a resource for phenotyping.

#### Cultivation of M_3_

The M_3 _progeny of identified mutants were grown for genotyping and for the determination of visible mutant phenotypes. Forward genetic mutant screening was performed on subsets of M_3 _families (16 seeds per single M_2 _individual) derived from EMS concentrations between 20 and 40 mM. These were cultivated under field conditions. Plants were scored for visible phenotypes during the seedling stage, tillering, awn emergence, time of heading and final maturity. All phenotypes were scored in reference to the parent cultivar 'Barke.'

### Genomic DNA isolation

Genomic DNA was prepared from young leaves after lyophilisation of tissue. Twenty to thirty milligrams of lyophilised leaf tissue (the remainder of each sample was stored as a backup) was milled in 2 ml tubes, and the DNA was extracted essentially according to a previously described procedure [49,50]. The DNA was subsequently transferred to 96-well plates and concentrations were measured with an FLX 800 Microplate Fluorescence Reader (384 square well plates; BIO-TEC Instruments, Inc.) using Hoechst 33258 blue dye (Sigma, Deisenhofen, Germany) according to the manufacturer's instructions (Version 04/2004). Aliquots of the DNA were diluted to a final concentration of 20 ng/μl for PCR and arranged in 2570 two-dimensional (2D) eight-fold pools [51] for mutation screening.

### Cel I-based mutation screening

#### Primers, PCR

Oligonucleotides for PCR were designed either directly using the Primer3 software [52] or via the program CODDLE (Codons Optimised to Discover Deleterious Lesions) [53]. Gene-specific primer design was either based on genomic or EST sequence information for the candidate genes. Unlabelled and identical primers labelled at the 5'end with the fluorescent dye IRD700 (forward) or IRD800 (reverse), respectively, were mixed and used for PCR amplification as follows: 3:2 (labelled : unlabeled) ratio for the 100 μM IRD700-labelled forward primer and 3:1 (labelled : unlabeled) ratio for the 100 μM IRD800-labelled reverse primer. Primers were designed to have melting temperatures between 60°C and 70°C. PCR amplification was carried out in a 30-μl volume containing 20 ng of individual pooled DNA, 1× buffer (Qiagen, Hilden, Germany), 0.2 mM dNTPs, 0.3 μM primers, and 0.03 U of Taq polymerase (Qiagen, Hilden, Germany). The PCR reactions were conducted using a thermal cycler (Applied Biosystems 9800 Fast Thermal Cycler, Foster City, USA) as follows: heat denaturation at 95°C for 3 min, followed by 8 cycles of touchdown PCR (94°C for 30 s, annealing at primer T_m _+3°C to T_m_+4°C for 30 s, decreasing -1°C per cycle, and extension at 72°C for 1 to 1.30 min [for 300- to 1.500-bp products]); 35 cycles of: heat denaturation at 94°C for 30 s, annealing at primer T_m _-4°C to T_m _-5°C for 30 s, decreasing -1°C per cycle, and extension at 72°C for 1 to 1.30 min; and final extension at 72°C for 10 min. The amplification step was followed by heteroduplex formation: inactivation and denaturation at 99°C for 10 min; and a re-annealing process of 70 cycles for 20 s at 70°C to 49°C, decreasing 0.3°C per cycle. The primers used for PCR amplification of DNA pools and sequencing are listed in Additional file 4.

#### CEL1 nuclease mismatch cleavage assay

After PCR amplification, samples were incubated for 45 min at 45°C in a 10-μl volume of 50-100 ng of DNA with 0.06 U of Surveyor CEL1 enzyme (Transgenomics, Omaha, USA) in a 10× buffer (10 mM HEPES (pH 7.0), 10 mM KCl, 10 mM MgCl_4*_7H_2_0, 0.002% Triton X-100 and 10 μg/ml BSA). *CEL1 *digestion was stopped by adding 5 μl of 75 mM EDTA (pH 8.0) followed by freezing (-20°C) of the probes for 30 min.

Precipitation of 30-μl samples in 96-well PCR plates was carried out by adding 5 μl of 3 M Na-Acetate (pH 5.2) and 75 μl of EtOH (99.8%), subsequent shaking for 15 - 20 min, and centrifugation for 30 min at 4470.6 × g. The supernatant was discarded by turning the plates facedown on top of a stack of filter paper and centrifuging for 1 min at 497 × g. The precipitated samples were washed in 100 μl of ethanol (75%) at room temperature (20 - 25°C) while centrifuging for 30 min at 4,470.6 × g. Removal of the supernatant was performed as described above. Finally, samples were dried for 20 min at room temperature, resuspended in 8 μl of formamide loading buffer (33% (w/v) deionised formamide (Roth, Karlsruhe, Germany), 25 mM Tris (pH 7.5), 25 mM EDTA and ~0.02% (w/v) bromphenol blue) with constant shaking (300 rpm) for 5-10 min, denatured for 5 min at 95°C and subsequently placed on ice until electrophoresis. Electrophoresis of the denatured DNA samples was performed (6.0% Long Ranger polyacrylamide slab gels, FMC Corporation, composition according to manufacturer's data; 1× TBE buffer: 89 mM Tris Base, 89 mM boric acid, 2 mM Na_2_EDTA_*_2H_2_0) at 1,500 V, 40 mA and 40 W settings on a Li-COR 4300 DNA Analyser (LI-COR Biosciences, Lincoln, NE). Gel images were visualised using the GelBuddy software [54,55] and inspected visually for the presence of cleavage products.

TILLING screens were conducted by using a CEL1-based heteroduplex analysis for mutation detection. The CEL1-based assay relies on using the LI-COR two laser/PA-slabgel system. The optimal mutation detection procedure and pool size for 'Barke' DNA was determined by performing test screenings using known mutations in the *Hv-eIF4E *gene [56] of the barley genotypes 'IGRI' and 'Franka.' Thereby, we found that for pooled DNA, a 1:10 dilution of CEL1 in a specific buffer solution combined with a 45 min digestion at 45°C provides an optimal ratio between the background and signal strength. The 8-fold arrangement was found to be most reliable, although mutations were still detectable in the tested 16-fold pools. To improve the mutation screening, the population was screened in a two-dimensional format. In each 96-well pool plate, the DNA of a total of 384 M_2 _individuals was arranged in row and column 8-fold pools. As each sample is present in two different pools, any mutation should be detected twice.

### DNA sequencing and sequence data processing

For confirmation of presumed mutated loci, amplicons of the respective target gene were generated from putative mutants by utilising the same PCR conditions as established for CEL I analysis. Amplicons were purified by ultrafiltration using a QIAvac 96 vacuum (Qiagen, Hilden, Germany), processed with a NucleoFast-96 PCR Plate (MACHEREY-NAGEL GmbH & Co. KG, Düren, Germany) and directly cycle-sequenced using the ABI Big Dye terminator v3.1 sequencing standard kit according to the manufacturer's protocol (Applied Biosystems, Foster City, USA). Sequencing reactions were resolved on a 96-capillary sequencing device (3730xl DNA Analyzer, Applied Biosystems, Foster City, USA). Sequences were aligned against the "wild-type" reference of the 'Barke' cultivar with Sequencher 4.6 software (Gene Codes, Ann Arbor, MI).

Genes were routinely analysed using the program CODDLE to obtain a gene model and to identify the region that would have the highest likelihood of being functionally affected by EMS mutagenesis. The software PARSESNP (Project Aligned Related Sequences and Evaluate SNPs) [57,58] was used to predict the severity of each identified mutation. If necessary, the Web version of the FGENESH program was used for gene structure prediction, and parameters were set for monocot plants [59]. The Conserved Domain Database (CDD) [60] accessible on the National Center for Biotechnology Information (NCBI) was used for detailed analysis of identified mutations, as it provides multiple sequence alignments and derived database search models that suggest protein domains conserved in molecular evolution [61,62].

### Statistical Analysis

Statistical analysis was conducted to investigate possible EMS dosage effects on genomic mutation frequencies, M_1 _germination and sterility, M_2 _chlorophyll mutant occurrence, and M_3 _seed yield. The *STATISTICA *data analysis software version 6 (StatSoft, Inc. (2001), http://www.statsoft.com) was used to conduct correlation analyses [(Pearson (r) and Spearman rank (r_s_)], tests of linear relationships [63] and one-factorial ANOVAs with the respective software protocols.

### How to access the TILLING population

The 'Barke' TILLING population was developed in the framework of the German GABI (Genome Analysis in the Biological system plant) program. Screening of the resource is not established as a service yet but can be performed on the basis of collaboration. For further information please contact Nils Stein, IPK Gatersleben.

## Authors' contributions

SG established the barley TILLING platform. PB and NS conceived the study. NS contributed to the design of the study. SG and NS wrote the manuscript. TK provided information on the *HvHox1 *gene, including the sequence and phenotypic data of additional barley mutants. UL was involved in the forward genetic screening of the mutant populations. All authors read and approved the manuscript.

## Supplementary Material

Additional file 1 **Additional figure 1 - Examples of chlorophyll-deficient seedling mutants in M_2_**. Supplemental figure showing chlorophyll-deficient seedling mutants. Mutants belonging to the unicolour sub-class: (A) albina, (B) xantha, and (C) viridis. Mutants belonging to the bicolour sub-class: (D) viridoalbina and (E) striata. The classification corresponds to [20]. Click here for file

Additional file 2 **Additional figure 2 - Frequency of mutant phenotype categories observed in M_2 _and M_3 _screenings**. This supplemental figure is an overview of the occurrence of different mutant phenotypes observed in the mutant populations. Mutant phenotypes observed in M_2 _and M_3 _derived from mutagenesis at 20 - 35 mM EMS were classified into six morphological categories. (A) In the M_2 _generation, approximately 20% of the 12,703 individuals showed a mutant phenotype vs. the 'Barke' wild-type. (B) In the M_3 _generation, 1,200 M_3 _families (16 plants per M_3 _line) were grown, and approximately 37% of the total M_3 _families displayed a mutant phenotype vs. the 'Barke' wild-type. Click here for file

Additional file 3 **Additional table 1 - A series of mutations in the *HvHox1 *gene identified by TILLING**. The additional table shows the position and mutation types identified in the *HvHox1 *gene. Click here for file

Additional file 4 **Additional table 2 - Sequences of primers used for TILLING of candidate genes**. This additional table specifies primers used for PCR amplification of DNA pools and sequencing. Click here for file
