# Supplementary figures and images for: TILLING in the two-rowed barley cultivar 'Barke' reveals preferred sites of functional diversity in the gene HvHox1
Source: BMC Res Notes. 2009 Dec 17;2:258. doi: 10.1186/1756-0500-2-258 (PMC2803498; doi:10.1186/1756-0500-2-258)

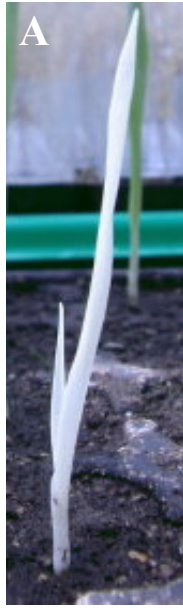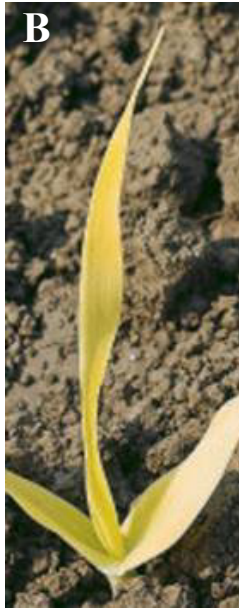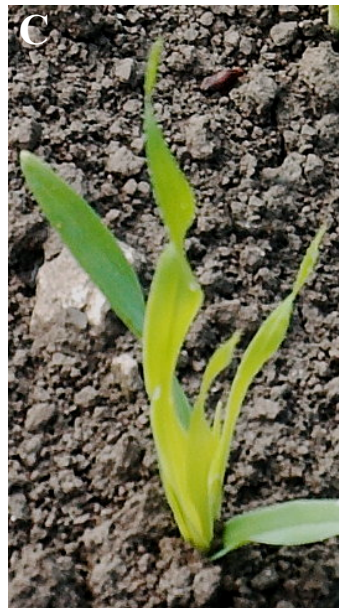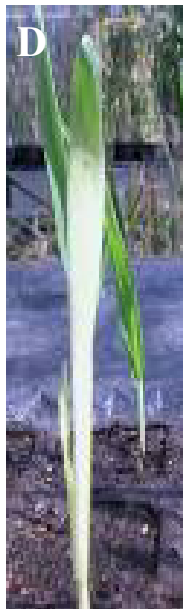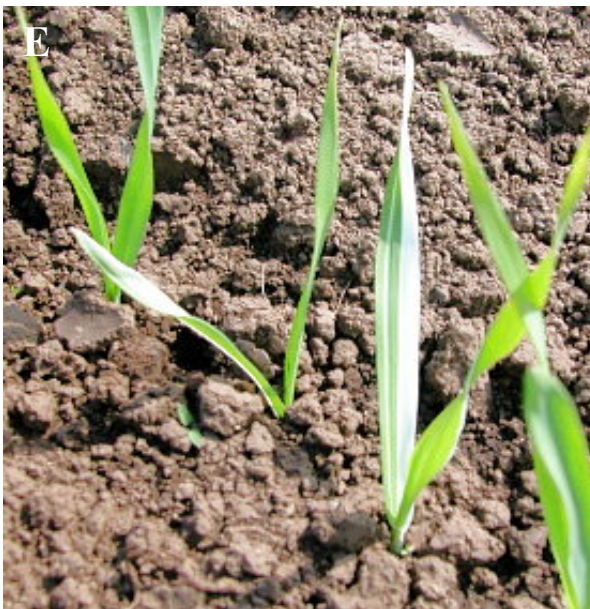

Supplement: Additional file 1 — Additional figure 1 - Examples of chlorophyll-deficient seedling mutants in M2. Supplemental figure showing chlorophyll-deficient seedling mutants. Mutants belonging to the unicolour sub-class: (A) albina, (B) xantha, and (C) viridis. Mutants belonging to the bicolour sub-class: (D) viridoalbina and (E) striata. The classification corresponds to [20]. [file 1756-0500-2-258-S1.PDF]

**A**

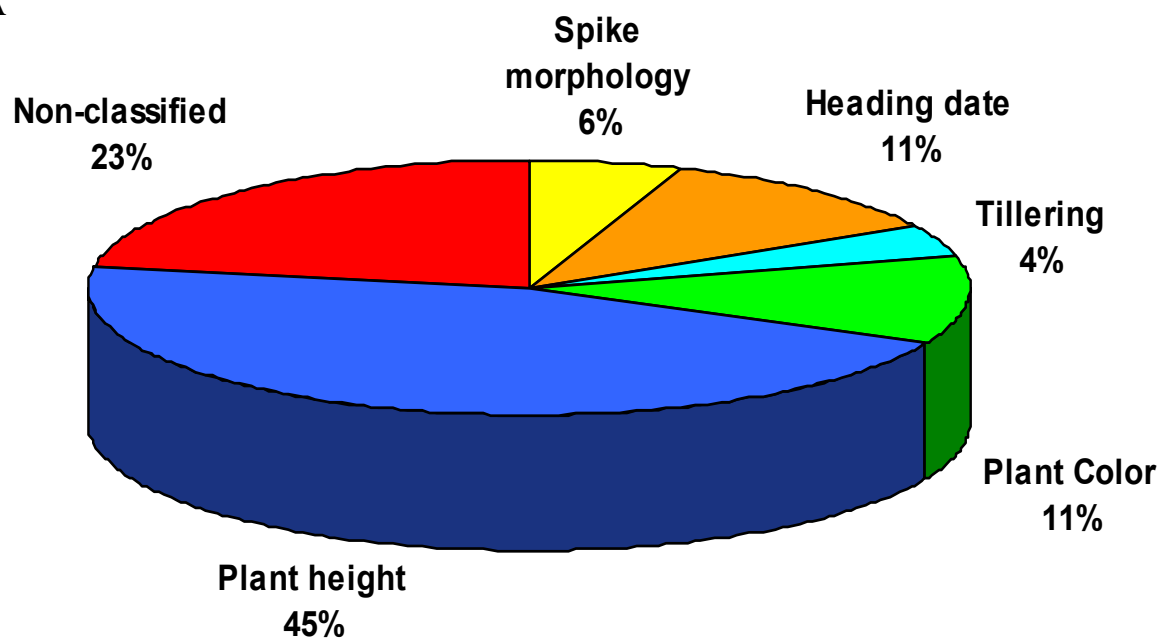

**B**

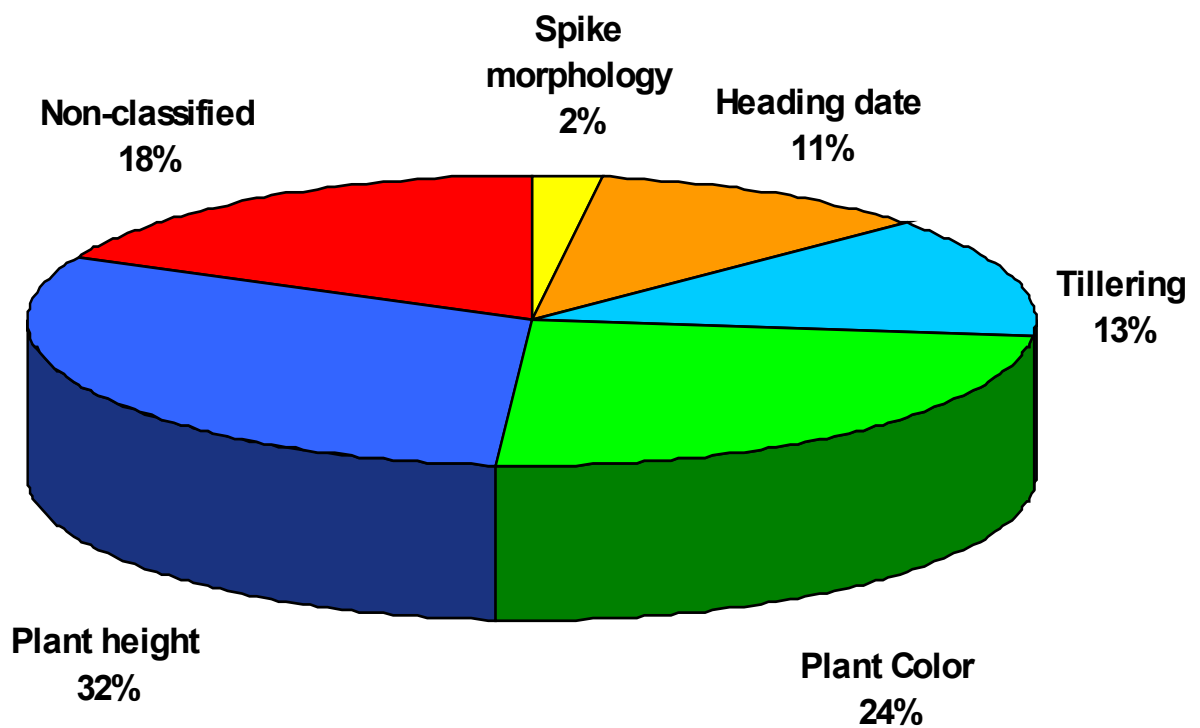

Supplement: Additional file 2 — Additional figure 2 - Frequency of mutant phenotype categories observed in M2 and M3 screenings. This supplemental figure is an overview of the occurrence of different mutant phenotypes observed in the mutant populations. Mutant phenotypes observed in M2 and M3 derived from mutagenesis at 20 - 35 mM EMS were classified into six morphological categories. (A) In the M2 generation, approximately 20% of the 12,703 individuals showed a mutant phenotype vs. the 'Barke' wild-type. (B) In the M3 generation, 1,200 M3 families (16 plants per M3 line) were grown, and approximately 37% of the total M3 families displayed a mutant phenotype vs. the 'Barke' wild-type. [file 1756-0500-2-258-S2.PDF]
