# Supplementary material for: TILLING in the two-rowed barley cultivar 'Barke' reveals preferred sites of functional diversity in the gene HvHox1
Source: BMC Res Notes. 2009 Dec 17;2:258. doi: 10.1186/1756-0500-2-258 (PMC2803498; doi:10.1186/1756-0500-2-258)
Supplement: Additional file 3 — Additional table 1 - A series of mutations in the HvHox1 gene identified by TILLING. The additional table shows the position and mutation types identified in the HvHox1 gene. [file 1756-0500-2-258-S3.DOC]

| **Additional Table 3: Series of mutations in the *HvHox1* gene identified by TILLING** | | | | | | | |
| --- | --- | --- | --- | --- | --- | --- | --- |
| Allele | Genotype | **Effect** | **Polarity change** | **HD-ZIP Domain** | **Spike phenotype** | **M_2_ plant** | **EMS dosage** |
| C0497T | homo | non-coding | - | - |  | 13527-1 | 50mM |
| C0562T | homo | non-coding | - | - |  | 12594-1 | 30mM |
| C0620T | hetero | non-coding | - | - |  | 876-1 | 30mM |
| C0625T | homo | non-coding | - | - |  | 1865-1 | 20mM |
| T0644C | homo | non-coding | - | - |  | 1248-1 | 20mM |
| C0782T | homo | Intron | - | - |  | 11821-1 | 35mM |
| G0847A | homo | Intron | - | - |  | 2997-1 | 25mM |
| G0856A | homo | Splice Junction | - | - | hex-v 6-rowed spike | 11910-1 | 35mM |
| G0900A | homo | synonymous | - | - |  | 11360-1 | 35mM |
| T0919A | homo | Cys to Ser | yes | no | normal 2-rowed spike | 2401-1 | 20mM |
| G0931A | hetero | Asp to Asn | yes | no | normal 2-rowed spike | 9587-1 | 30mM |
| G0935A | hetero | Met to Thr | yes | no | normal 2-rowed spike | 10759-1 | 30mM |
| G0945A | homo | synonymous | - | - |  | 374-1 | 25mM |
| C0956A | hetero | Gly to Glu | yes | no | normal 2-rowed spike | 4674-1 | 30mM |
| C0968T | homo | Pro to Leu | no | yes | normal 2-rowed spike | 10872-1 | 30mM |
| C0969A | homo | synonymous | - | - |  | 13604-1 | 50mM |
| G0996A | homo | synonymous | - | - |  | 2564-1 | 20mM |
| G1039A | homo | Glu to Lys | yes | yes | lateral spikelets enlarged and pointed | 3930-1 | 25mM |
| A1044T | hetero | synonymous | - | - |  | 1648-1 | 20mM |
| C1078T | hetero | synonymous | - | - |  | 1503-1 | 20mM |
| T1079A | homo | Leu to Gln | yes | yes | hex-v 6-rowed spike | 8408-1 | 25mM |
| G1115A | homo | Arg to His | no | yes | int-d 6-rowed spike | 11657-1 | 35mM |
| G1140A | homo | synonymous | - | - |  | 10109-1 | 30mM |
| C1188T | hetero | synonymous | - | - |  | 3548-1 | 25mM |
| C1271T | homo | Intron | - | - |  | 10268-1 | 30mM |
| C1280T | hetero | Intron | - | - |  | 4367-1 | 30mM |
| T1421C | hetero | synonymous | - | - |  | 9955-1 | 30mM |
| C1442T | hetero | synonymous | - | - |  | 786-2 | 30mM |
| G1471A | homo | Gly to Glu | yes | no | normal 2-rowed spike | 13213-1 | 40mM |
| G1484A | homo | synonymous | - | - |  | 12674-1 | 40mM |
| G1491A | homo | Gly to Ser | no | no | normal 2-rowed spike | 11580-1 | 35mM |
