# Supplementary material for: TILLING in the two-rowed barley cultivar 'Barke' reveals preferred sites of functional diversity in the gene HvHox1
Source: BMC Res Notes. 2009 Dec 17;2:258. doi: 10.1186/1756-0500-2-258 (PMC2803498; doi:10.1186/1756-0500-2-258)
Supplement: Additional file 4 — Additional table 2 - Sequences of primers used for TILLING of candidate genes. This additional table specifies primers used for PCR amplification of DNA pools and sequencing. [file 1756-0500-2-258-S4.DOC]

| Additional Table 4: Sequences of primers used for TILLING of candidate genes | |
| --- | --- |
| **Primer name** | **Sequence (5´ → 3´)** |
| *HvCO1*-F | GCA AGT CAC AAG GCC ACC T |
| *HvCO1*-R1 | GCT GTT GTT GAC GGA ATC TG |
| *Mlo9*-F1 | AGC AAA CCA GAC ACA CAG CAG CGT ACC |
| *Mlo9*-R1 | GCA AAG GCT CAC TTT GAG ACG GCT TAG |
| *Mlo9*-F2 | CAT TTG TCG CAA AAC AGC AAG TTC GAC |
| *Mlo9*-R2 | TTG TCT CAT CCC TGG CTG AAG GAA AAA |
| *HveIF4E*-F1 | CTA GCA AGT TGA ATG TTG GAG C |
| *HveIF4E*-R1 | GAA TAC ATA CCT GAG CAG TTT C |
| *HveIF4E*-F2 | GTT TGT ATC ATT TCC AGC CGA |
| *HveIF4E*- R2 | TGC ATC CAA GAG TAG GCG GGC |
| *HvDnaJ-like*-F1 | TTG CCG GGC TAT ATC TGA GGA TT |
| *HvDnaJ-like*-R1 | GCC TCG GTT ATC TGC TTG AAC CT |
| *HvHox1*-F1 | AAG CAT GGA CAA GCA TCA GCT CTT TGA |
| *HvHox1*-R1 | GGC AGC AGC TAT CTC GGC TAT TTT ATG G |
| *HvHox1*-F2 | TCA ATG CTA ATG TGG AGA GTA AAT AAA |
| *HvHox1*-R2 | ATA TGT TTG TCA GTA AGC ATG TTC AAG |
| *HvCIGR2*-F1 | CTG TCA GTG CTT CAT GCG TC |
| *HvCIGR2*-R1 | CTC GAA GAT GGC CGT GTA GT |
| *HvCIGR2*-F2 | CCA TGC AGT GAT TAG CGT GT |
| *HvCIGR2*-R2 | GAC CGA AAC CAT CTT CCT CA |
